# 

**Published:** 1857-08

**Authors:** 


### RECEIPTS ON SUBSCRIPTIONS,

UP TO AUG. 1ST, 1857.

### ILLINOIS.

| | | | |
|---|---|---|---|
| Dr. A. W. Abbott, | VOL. | 6, | $2.00 |
| " O. Everett, | " | 4, 5&6, | 6.00 |
| " D. B. Jewell, | " | 4, 5&6, | 6.00 |
| " J. Welch, | " | 6, | 2.00 |
| " T. B. Dever, | " | 6, | 2.00 |
| " J. R. Ebere, | " | 6, | 2.00 |
| " J. C. Jewett, | " | 6, | 2.00 |
| " L. D. Glazebrook, | " | 5, | 2.00 |
| " O, C. Denslow, | " | 4, | 2.00 |
| " G. W. Kittell, | " | 6, | 2.00 |
| Drs. Robinson & Zearing, | VOL. | 5, | 2.00 |
| " Brackett & Buckly, | " | 6, | 2.00 |
| Dr. A. Mahan, | " | 6, | 2.00 |
| " O. Q. Herrick, | " | 6, | 2.00 |
| " Jos. H. Faris, | " | 5, | 2.00 |
| " J. S. Bullock, | VOL. | 5&6, | 4.00 |
| " J. F. Spilman, | " | 6, | 2.00 |
| " J. N. Allen, | " | 5&6, | 4.00 |
| " — See, | " | 6, | 2,00 |
| " J. T. Pearman, | " | 6, | 2.00 |
| " John Bowman, | " | 6, | 1.00 |
| " B. Wilson, | " | 6, | 2.00 |
| " D. M. Marshall, | " | 6, | 2.00 |
| " W. H. Heller, | " | 5&6, | 4.00 |
| " Robt. Pice, | " | 6, | 2.00 |

### IOWA.

| | | | |
|---|---|---|---|
| Dr. A. E. Smith, | VOL. | 6, | $2.00 |
| " Lorenzo Bents, | " | 6, | 2.00 |
| " J. S. M. C. Van Nus, | " | 6, | 2.00 |
| " E. Hollingsworth, | " | 5, | 2.00 |
| " L. R. McCullough, | " | 6, | 1.00 |
| " T. C. Jennings, | " | 6, | 1.00 |
| " J. N. B. Elliott, | " | 6, | 2.00 |
| " H. A. May, | " | 6, | 1.00 |

### INDIANA.

| | | | |
|---|---|---|---|
| Dr. C. Palmiter, | VOL. | 6, | $2.00 |
| " W. H. Gifford, | " | 5, | 2.00 |
| " J. N. Wardlaw, | " | 5, | 2.00 |
| " B. F. Newland, | " | 6, | 2.00 |
| " Thos. Newkirk, | | 5&6, | 4.00 |
| " G. Newland, | " | 6, | 2.00 |

### WISCONSIN.

| | | | |
|---|---|---|---|
| Dr. John Wiley, | VOL. | 6, | $2.00 |
| " C. Nettleton, | " | 6, | 2.00 |
| " J. M. Ball, | | 2&3, | 3.00 |
| " L. B. W. Johnson, | | 5&6, | 4.00 |

### MISSOURI.

| | | | |
|---|---|---|---|
| Dr. R. B. Ray, | VOL. | 6, | $2.00 |

